# Validating the Edinburgh Postnatal Depression Scale as a screening tool for postpartum depression in Kathmandu, Nepal

**DOI:** 10.1186/s13033-016-0102-6

**Published:** 2016-10-21

**Authors:** Babu Ram Bhusal, Nisha Bhandari, Manisha Chapagai, Tania Gavidia

**Affiliations:** 1 Ministry of Health, Government of Nepal, Kathmandu, Nepal; 2 Faculty of Maharajgunj Nursing Campus, Institute of Medicine, Tribhuwan University, Kathmandu, Nepal; 3 Department of Psychiatry and Mental Health, Maharajgunj Medical Campus, Institute of Medicine, Kathmandu, Nepal; 4 Curtin University, Bentley, WA Australia

**Keywords:** Validation, Postpartum depression, Edinburgh Postnatal Depression Scale (EPDS), Kathmandu, Nepal

## Abstract

**Background:**

Edinburgh Postnatal Depression Scale (EPDS) is considered well accepted screening tool for postpartum depression (PPD). The objective of the study was to validate the EPDS as a screening tool for postpartum depression in Kathmandu, Nepal.

**Methods:**

A hospital based cross sectional study using EPDS was conducted among 346 mothers between 4 and 14 weeks of postpartum period. All the participants were examined by psychiatrist for possible clinical PPD diagnosis using International Classification of Disease tenth revision (ICD-10). Sensitivity, specificity, positive predictive value and negative predictive value were calculated for validation of EPDS. The best cut off point for Nepalese version of EPDS was identified and area of the receiver operating characteristics curve was calculated.

**Results:**

The overall prevalence of PPD was 17.1 %.The sensitivity, specificity, positive predictive value and negative predictive value of the Nepalese version EPDS was found to be 92, 95.6, 77 and 99.3 % respectively. The best cut-off point of EPDS for screening of PPD was found to be 12/13 and the area of the curve was 0.98 (95 % CI 0.970–0.994, p = 0.001).

**Conclusions:**

The prevalence of PPD is not that far from the previous studies of Nepal. Nepali version of EPDS was acceptable and the study demonstrates good validity, thus EPDS can be used as valid screening tool for PPD for early detection, prompt treatment and to prevent possible consequences.

## Background

Postpartum depression is defined as an episode of non-psychotic depression according to standardized diagnostic criteria with onset within 1 year of childbirth [1]. Approximately 50–80 % of women suffered from maternal sadness in the puerperal period, with about 20 % of those women develop postpartum depression [2]. However, there is a wide variation in prevalence of postpartum depression from almost affecting 8–50 % of postnatal mothers [3–7] in different countries. The prevalence of PPD is estimated to be higher in low and middle income countries than for high income countries primarily as many women don’t seek help or tell others about their feelings [8–12]. British Medical Bulletin, 2012 reported that low income countries such as Nepal and Malaysia had prevalence of PPD below 13 % and in other low income countries it ranges from 13 to 50 % [7]. The studies conducted in Nepal showed the prevalence of PPD ranging from 4.9 to 19.4 % [13–15]. The differences in the magnitude of prevalence may be due to transcultural variations and interpretation of symptoms and also socio-economic variables [16].

Postpartum Depression can have several consequences which includes maternal death due to suicide [17], the mother infant relationship [18], psychological development of child [19], infant nutrition [20] and infant growth [21]. Postpartum depression is infrequently diagnosed and indeed treated, though its significant incidence and morbidity [22]. Depressive symptoms are dismissed as they mimic normal physiologic changes [23–25] and formerly used to be ignored in health care services [26]. The effects of postnatal depression on the mother, her marital relationship, and her children make it an important condition to diagnose, treat and prevent [1].

The routine use of screening scales for the purpose of identifying symptoms of depression is an effective, simple and economical way to identify women at risk [27]. There are some instruments designed to measure PPD which includes: The Edinburgh Postnatal Depression Scale [28], Beck Depression Scale (BDI) [29], Zung Self Rating Scale [30], the Kessler Psychological Distress Scale (K10) [31] and the Self-Reporting Questionnaire (SRQ20) [32]. In the postpartum period, the EPDS has been the most widely used scale to identify postpartum depression [4, 33–35]. Cox, Halden and Sagovsky, in 1987 identified EPDS as fully acceptable tool for detection of PPD with simple method of scoring. It is a 10-item self-report questionnaire in which women are asked to rate how they have felt in the previous 7 days and each question is scored 0–3 resulting in total score range of 0–30. The tool has satisfactory sensitivity and specificity and was also sensitive to change in severity of depression over time [36].

With the development of EPDS, it has been validated and used in many countries [4, 34, 35, 37, 38]. The sensitivity observed in the validation studies presented variations ranging from 65 to 100 %, while the specificity with 49–100 % in range with different cut-off points [4, 34, 35, 37, 39, 40]. In the validation studies done in Nepal there is also wide range of variation in sensitivity with 68–100 % whereas less variation in specificity 93–94 % [13, 41]. The great variability of results among the different studies was due to variations in methodology used, diagnostic criteria and different weeks of postpartum [4, 34, 35, 37, 39–41].

Validation of EPDS is an important endeavor that provides reliability of the instrument when next version other than original English is used. As there is wide variation in sensitivity of EPDS in various validation studies conducted in Nepal, this study aimed to validate original English version of EPDS into Nepali version in order to measure the accuracy of the EPDS to screen postpartum depressive symptoms.

## Methods

### The aim, design and setting of the study

Descriptive cross-sectional design was used to validate the EPDS. The study was conducted at the Tribhuvan University Teaching Hospital (TUTH) in Kathmandu, Nepal. It is the tertiary level facility situated in capital of Nepal.

### Participants

The study population consisted of 4–14 weeks postpartum mothers attending child immunization clinic. Women suffering from severe medical illness, personal and family history of mood disorder were excluded as physical symptoms (Insomnia, anorexia, decreased concentration and pain etc.) could mimic those of depression and may bias the sample. Women with mental retardation were also excluded because of the probability of interference in the process of informed consent and even also during the data collection.

### Sample size

The sample was chosen by systematic random sampling method from those who registered in the clinic before immunization of children. The required sample size was calculated from

$${\text{n}} = {\text{Z}}^{2} {\text{pq}}/{\text{d}}^{2}$$

Z = 1.96, P = 8 % = 0.08, q = 1 − p = 0.92, allowable error L = 3 % = 0.03.

The prevalence estimated by various study in Nepal was 12 and 5 % respectively [13, 14]. Hence, prevalence of 8 was estimated by taking an average. The sample size was 346 at 95 % confidence limit and power of 80 with 10 % of non-response rate.

### Data collection tools and technique

The EPDS was used to detect depression as a screening tool. The EPDS is a ten item self-report questionnaire. Items 1 and 2 assess anhedonia; item 3: self-blame; item 4: anxiety; item 5: fear or panic; item 6: inability to cope; item 7: difficulty sleeping; item 8: sadness; item 9: tearfulness and item 10: self-harm ideas. Responses are scored as 0, 1, 2 and 3 according to the increasing severity of the symptoms. Total score is calculated by adding each score of the each 10 items. The value of score can range from 0 to 30 [27].

Forward translation from original English version of EPDS to Nepali version was done by two bilingual psychiatrist of TUTH. Then backward translation from Nepali version to English version was done by a group of professional translators. The back translated English version was reviewed by native English speaker. In order to make scale sensitive to culture and social strata, conceptual and linguistic equivalence was considered during the translation process. The principal investigator pre-tested the tool in Thapathali Maternity Hospital by using interview technique. The reliability of the Nepalese version EPDS scale was determined by reliability index i.e. Cronbach’s alpha and found to be 0.74 which showed the acceptable range of reliability. The tool was well accepted and did not demand any modification. The data collection was then carried out by principal investigator by using EPDS as a screening tool. After administration of EPDS, the diagnostic interview was taken by a psychiatrist using ICD-10 as gold standard. The ICD-10 diagnostic protocol was translated into Nepali language. The psychiatrist was blinded to the initial screening results. The diagnostic interview was taken in same day to avoid possible fluctuation of mental status in the postpartum period. The structured questionnaire was used to collect the demographic information. All the participants agreed to participate in the study and none of the respondent withdrew.

### Data management and analysis

Data was compiled and checked for completeness and entered into EPI-INFO version 3.5.1. The entered data were exported to Statistical Package for Social Science (SPSS) version 20. Descriptive analysis was done for demographic variables. The sensitivity, specificity, positive predictive value and negative predictive value of the translated version of EPDS were obtained to determine the validity of EPDS. The identification of best cut off score was done by cross tabulating the value of sensitivity and 1-specificity for each point. The accuracy of the screening tool at best cutoff point was identified from ROC curve by computing area under curve.

### Ethical consideration

The study was approved by Institutional Review Board of Institute of Medicine, Tribhuvan University. The purpose of the study was explained and written consent was taken from all the participants.

## Results

### Socio-demographic characteristics of the study population

The socio- demographic data were described in terms of age, ethnicity, educational status, occupation and parity. Of all participants, mean age was 22.75 (SD = 4.51) with more than half (51 %) being Brahmin/Khsetri in ethnicity. The majority of respondent (57 %) completed their higher secondary and above. About half of the participants (49 %) were engaged in domestic/household work. Fourty-four percent respondent had only one child (Table 1).

**Table 1:** Socio-demographic characteristics of study population

Variables	Frequency (n = 346)	Percentage
Age of mother		
<20	29	8.4
20–25	162	46.8
26–30	93	26.9
31–35	54	15.6
>35	8	2.3
Ethnicity		
Dalit	23	6.6
Janajati	120	34.7
Madhesi	14	4
Muslim	3	0.9
Braman/Kshitri	177	51.2
Others/Thakuri/Sanyasi	9	2.6
Educational status		
Illiterate	5	1.4
Able to read and write	13	3.8
Primary	16	4.6
Lower secondary	21	6.1
Secondary	94	27.2
Higher secondary	105	30.3
Bachelor and above	92	26.6
Occupation		
Agriculture	5	1.4
Labor/wage	30	8.7
Domestic work	170	49.1
Service	75	21.7
Business	61	17.6
Others	5	1.4
Parity		
One	153	44.2
Two	136	39.3
Three	47	13.6
More than three	10	2.9

### Prevalence of PPD

Out of 346 participants, 59 were found to be suffering from PPD by EPDS at the cutoff point of 12/13. Hence, the prevalence rate of PPD was found to be 17.1 % (Table 2).

**Table 2:** Prevalence of postpartum depression

Variables	Frequency (n = 346)	Percentage
No depression	287	82.9
Depression	59	17.1

### Validation of EPDS

The EPDS scores were cross tabulated with the actual PPD status of the participants from diagnostic interview. From this the corresponding sensitivity (proportion of depressed mothers according to ICD-10 criteria that were correctly identified by EPDS), specificity (proportion of non-depressed mothers correctly identified as such by EPDS), positive predictive value (proportion of true positives among all positives identified by EPDS) and negative predictive value (proportion of true negatives among all negatives identified by EPDS) were calculated. These are the measures of determining the validity of the scale for screening of the PPD (Table 3).

**Table 3:** Validation of EPDS

Diagnosis by Gold standard test (ICD-10)			Total
	Positive (+ve)	Negative (−ve)	
Screening by EPDS			
Positive (+ve)	46 (true positive/TP)	13 (false positive/TF)	59
Negative (−ve)	4 (false negative/FN)	283 (true negative/TN)	287
Total	50	296	

Here, sensitivity = TP/TP + FN = 46/46 + 4 = 0.92 = 92 %

Specificity = TN/TN + FP = 283/283 + 13 = 95.6 %

Positive predictive value (PPV) = TP/TP + FP = 46/46 + 13 = 0.77 = 77 %

Negative predictive value (NPV) = TN/TN + FN = 287/287 + 4 = 99.3 %

The sensitivity between 80 and 90 % is considered quite well and the screening test can correctly identify those who have depression. Similarly, specificity 95 % is also considered quite good to identify correctly those who do not have depression. Hence, the EPDS is identified as the valid tool to measure the PPD in Nepalese context in this study.

### The EPDS cutoffs point

Screening tests use cutoff points to assess the threshold above which the tests are reliably accurate to identify the presence of given condition. In case of EPDS, the sum total score are computed against the clinical diagnosis and the levels of sensitivity (true positive) and 1-specificity (false positive) were assessed for each score (Table 4).

**Table 4:** Sensitivity and 1-specificity of Nepalese version of EPDS compared with ICD-10 results

	Sensitivity	1-specificity
≥10	0.980	0.125
≥11	0.940	0.088
≥12	0.920	0.044
≥13	0.880	0.041
≥14	0.780	0.030
≥15	0.680	0.014

It can be noted from above table EPDS cutoff levels of 12 and 13 are close in their sensitivity (true positive) and 1-specificity (false positive) values. Hence, the cutoff point of the screening test was found at 12/13.

### The ROC curve for the EPDS screening test

**Fig. 1:**
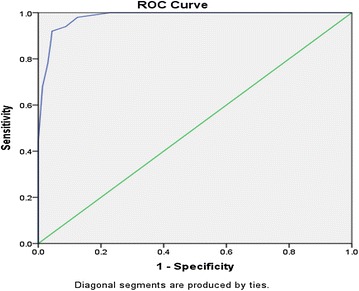
ROC curve for the screening of PPD by EPDS scale. Receiver operating characteristic curve for the EPDS screening test, demonstrating area under curve at chosen cutoff level i.e. (12/13). The area under curve was 0.982. Thus the Nepalese version of EPDS had accuracy of 98.2 % (95 % CI 0.970–0.994, p = 0.001) in screening for cases of PPD with the cutoff at 12/13. Hence, this cut off level can be considered optimum to define threshold level for depressive symptoms

To determine whether this cutoff level should actually be considered as the cutoff for Nepalese version of EPDS in screening the PPD status, the receiver operating characteristics curve (ROC) was also computed (Fig. 1).

## Discussion

The present study showed the overall prevalence of 17.1 % with cutoff point of 12/13.The prevalence of the study was lower than the prevalence found by Budathoki (19.4 % at 6 weeks and 22.2 % at 10 weeks of postpartum) which was done in Kathmandu Medical College Teaching Hospital and Dhading District Hospital with cutoff point of >13 [15], whereas, the prevalence was higher than in the study done by Regmi (12 %) in tertiary level hospital [13]. The findings lie in between these two studies. The minor alteration in prevalence might be due to differences in the study methodology and diagnostic criteria and also due to variation in the sample size. The prevalence not essentially contradict with that of the other countries which includes Thailand (10 %) [35], New Zealand (17.1 %) [40], Pakistan (16.4 %) [42] and United Kingdom (10–15 %) [43]. The prevalence of this study was not that far with the studies of other countries though there is variation in social structure, different educational status, social support, economic status, availability of health services, parenting methods and more. However, several studies have found high rate of depression in low and middle income countries [8–12].

The sensitivity, specificity, positive predictive value and negative predictive value of the Nepalese version EPDS found by this study was 92, 95.6, 77 and 99.3 % respectively at threshold level 12/13. The original English version of EPDS by Cox, identified sensitivity and specificity of 86 and 78 % respectively with threshold level above 12/13 [36]. The study done for validation of Nepalese version of EPDS by Regmi had shown sensitivity of 100 %, specificity of 92.6 %, positive predictive value of 41.6 % and negative predictive value of 100 % with cutoff point 12/13[13]. Also, an another study done in Nepal in 1999, had detected sensitivity of 68.3 %, specificity of 93.8 %, positive predictive value of 65 % and negative predictive value of 94.64 % at cut off point of 12/13 [41]. The hospital based study done in India, revealed the sensitivity of 92 % and specificity of 85 % [44] while the study done in urban slum of Karachi, Pakistan had shown sensitivity of 79 % and specificity of 74 % [42]. This study showed better psychometric properties than in the original English version EPDS by Cox et al., earlier Nepali language EPDS validation studies, studies from neighboring countries like India and Pakistan. This might be due to difference in methodology, translation process, sample size, diagnostic interview and timing of testing. The participants who visit health facilities of urban area are well educated and can have good understanding of translated version. In our study, more than two-third of participants were educated up to secondary level and above, so may have the greater emotional literacy and this might be the reason for good psychometric properties. However, the systemic study of Shrestha and colleagues found psychometric properties from low-and middle-income countries to be lower than that for the original English version of EPDS [45].

Having well defined cut off point appropriate at local setting is essential for accurate detection and estimation of PPD burden in the country. The cutoff point of Nepalese version of EPDS identified by this study was 12/13 which is similar with the previous studies done in Nepal by Regmi et al. and Nepal et al. [13, 41]. The cutoff score was also somewhat equivalent with the study from low and lower-middle-income countries like India (11/12) [44], Pakistan (13/14) [42], Mongolia (12/13) [46], Ghana (10/11) [47] and Zimbabwe (10/11) [48]. But the cutoff point was higher than the studies done in Ethiopia (5/6) [49], Nigeria (8/9) [50], Vietnam (3/4) [51] and Malawi (4/5) [52]. The variation in cutoff point score seen in different validation studies may be due to social and economic diversity, cultural norms and heterogeneities in sample characteristics. The higher EPDS cutoff score ensures the screening test has the best performance by identifying most cases so that a diagnostic or/and therapeutic intervention could be offered.

Culture sensitive translation, empirical validation, better psychometric properties and consistency of findings with national and international literature suggested that the Nepalese version of EPDS is valid instrument to screen the postpartum depressive symptoms. On the other hand, the study exhibited some limitations to be used in all settings of Nepal. As the sample was drawn from only one tertiary level hospital, the findings from this study may be limited to clinical setting and might have less generalizability in the culturally and socially diverse context.

## Conclusion

From our result and those obtained from other studies it can be concluded that Nepalese version of the EPDS can be successfully used to screen the postpartum depression in Nepalese women. These findings might be the inferences for the future planning and implementation of maternal mental health programs and policies in low and lower-middle-income countries.

## Abbreviations

CI: confidence interval
EPDS: Edinburgh Postnatal Depression Scale
FN: false negative
FP: false positive
ICD-10: International Classification of Diseases (Version 10)
OR: odds ratio
PPD: postpartum depression
NPV: negative predictive value
PPV: positive predictive value
ROC: receiver operating characteristics curve
SD: standard deviation
TN: true negative
TP: true positive
TUTH: Tribhuvan University Teaching Hospital
